# The Benefits of Music Listening for Induced State Anxiety: Behavioral and Physiological Evidence

**DOI:** 10.3390/brainsci11101332

**Published:** 2021-10-09

**Authors:** Binxin Huang, Xiaoting Hao, Siyu Long, Rui Ding, Junce Wang, Yan Liu, Sijia Guo, Jing Lu, Manxi He, Dezhong Yao

**Affiliations:** 1 The Clinical Hospital of Chengdu Brain Science Institute, MOE Key Laboratory for Neuroinformation, University of Electronic Science and Technology of China, Chengdu 611731, China; huangbinxin@std.uestc.edu.cn (B.H.); dyao@uestc.edu.cn (D.Y.); 2 School of Life Science and Technology, Center for Information in Medicine, University of Electronic Science and Technology of China, Chengdu 611731, China; siyu.long@std.uestc.edu.cn (S.L.); rui.ding@std.uestc.edu.cn (R.D.); 202022140301@std.uestc.edu.cn (J.W.); 2018140601017@std.uestc.edu.cn (Y.L.); sjguo@std.uestc.edu.cn (S.G.); 3 Department of Neurology, West China Hospital, Sichuan University, Chengdu 610041, China; sherryhao@wchscu.cn; 4 Research Unit of NeuroInformation, Chinese Academy of Medical Sciences, 2019RU035, Chengdu 611731, China; 5 School of Electrical Engineering, Zhengzhou University, Zhengzhou 450001, China

**Keywords:** state anxiety, musical modulation, emotion, power spectral density, functional connectivity

## Abstract

Background: Some clinical studies have indicated that neutral and happy music may relieve state anxiety. However, the brain mechanisms by which these effective interventions in music impact state anxiety remain unknown. Methods: In this study, we selected music with clinical effects for therapy, and 62 subjects were included using the evoked anxiety paradigm. After evoking anxiety with a visual stimulus, all subjects were randomly divided into three groups (listening to happy music, neutral music and a blank stimulus), and EEG signals were acquired. Results: We found that different emotional types of music might have different mechanisms in state anxiety interventions. Neutral music had the effect of alleviating state anxiety. The brain mechanisms supported that neutral music ameliorating state anxiety was associated with decreased power spectral density of the occipital lobe and increased brain functional connectivity between the occipital lobe and frontal lobe. Happy music also had the effect of alleviating state anxiety, and the brain mechanism was associated with enhanced brain functional connectivity between the occipital lobe and right temporal lobe. Conclusions: This study may be important for a deep understanding of the mechanisms associated with state anxiety music interventions and may further contribute to future clinical treatment using nonpharmaceutical interventions.

## 1. Introduction

State anxiety is a situation-specific form of anxiety [1] that subjects experience at a particular moment. For example, it can be increased in the presence of an anxiogenic stimulus under examination stress and before an interview. However, long-term state anxiety often elicits somatic symptoms (e.g., nervous system neuropathy [2], cardiovascular disease [3], breathing disorder and sleep disturbances [4]). Anxiety that persisted for a long time was a risk factor for depression [5] and resulted in a higher risk of suicide [6]. 

Diverse means are used to reduce anxiety, including pharmacological interventions [7] and nonpharmacological treatments (e.g., exercise, meditation [8], cognitive-behavioral psychotherapy [9] and music therapy [10]), although music interventions are economical, noninvasive and convenient and have been shown to have considerable potential for clinical application. For example, music intervention was an option to reduce anxiety during pregnancy [10]. Listening to music may also help patients with severe disease to ameliorate anxiety and reduce antimicrobial use [11]. Further investigation revealed that neutral music and happy music mitigate anxiety [12,13,14]. Even though these studies have indicated that music interventions relieve anxiety levels before particular situations, the type of music that works best and the brain mechanisms evoked by music interventions are still far from clear.

The basolateral amygdala (BLA) and ventral hippocampus (vHPC) can modulate anxious states in rats, and optogenetic inhibition of the BLA to the vHPC reduces avoidance behavior to produce anxiolytic effects [15]. Gamma oscillations may provide windows for coupling BLA activity to hippocampal and prefrontal inputs [16] and the abnormal dynamic functional networks in gamma band during early emotion processing enabled negative emotion recognition [17]. However, given the purpose of evolutionary fitness and fear [18], the neural circuits of anxiety might be widely distributed. In healthy volunteers, a significant blood flow increase in the bilateral temporal poles was observed during anticipatory anxiety, which was implicated in a lactate-induced anxiety attack in patients with panic disorder [19]. Additionally, a sustained threat context that stimulated contextual anxiety increased cerebral blood flow in the right hippocampus, mid-cingulate gyrus, thalamus, bilateral ventral striatum and occipital cortex [20]. In another study, disturbing pictures rated as high anxiety provoked activation in the occipital cortex and both amygdalae [21]. In an integrated view, stress-induced anxiety is closely linked to activity in the temporal lobes and occipital gyrus [22].

Music signals from the auditory cortex and the hypothalamus to the amygdala and the medial prefrontal cortex (PFC) regions are associated with emotional behavior processing [21,22]. Some evidence demonstrates that music can activate several brain regions with the limbic system, such as the hippocampus, amygdala and cingulate [23,24]. Notably, however, emotional processing of music was not exclusive to subcortical areas, and the cerebral cortex was also involved in modulation [25]. The superior temporal gyrus is known to be involved in auditory processing, where the characteristic response is mainly focused on the gamma band [26]. Indeed, a simple steady-state auditory click stimulation paradigm at gamma frequency (~40 Hz) has been reproducibly shown to reduce entrainment as measured by electroencephalography (EEG) in patients [27,28] and listening to music generating perceptual phenomenon was associated with different changes in gamma power (35–45 Hz) [29]. Acoustic tomography allows us to transform acoustic information into visual representations [30], and gamma is linked to the transformation of sensory signals along feedforward pathways [31]. In addition, an imagined music performance increased connectivity of the supplementary motor area with widespread regions in the brain, including the sensorimotor cortices, posterior temporal cortex and occipital cortex [32]. The gamma band played a vital role to assess the effectiveness of neurofeedback training in the context of clinical and social neuroscience [33]. Notably, these brain regions are highly related to anxiety. However, the precise regulatory mechanism needs to be further explored.

To study the neurocognitive basis of state anxiety, we first applied neutral and happy music as measures for a state anxiety intervention. A blank stimulus was selected for the control group. Subsequently, we examined the basic distinctions between two different emotional types of music. Considering individual differences, we also correlated trait personality and trait coping style with state anxiety.

### 1.1. Participants

In total, 68 healthy adults who were right-handed and had no color blindness, no color weakness and normal or corrected-to-normal vision, with normal hearing and no history of dyslexia were recruited at the University of Electronic Science and Technology of China. All participants without anxiety under the Self-rating Anxiety Scale had only a primary music education, had no neurological, psychiatric or other health problems and had not been on medication in the previous three months. In the experiment, the data from 6 participants were excluded from electroencephalogram (EEG) analysis due to excessive noise and excessive head motion during EEG acquisition. Thus, the final sample consisted of 62 participants (32 males, 30 females; M ± SD age, 23.339 ± 0.163 y).

The State-Trait Anxiety Inventory (STAI), the Big Five Inventory (BFI) and the Trait Coping Style Questionnaire (TCSQ) were completed before the experiment. State anxiety was induced in all participants using negative pictures and videos. Then, the participants received three types of stimulation, and their EEG signals were recorded.

### 1.2. Stimulation

#### 1.2.1. International Affective Picture System (IAPS)

In this case, 100 pictures were selected based on their normative valence ratings from the International Affective Picture System (IAPS), a collection of standardized photographic materials [34,35]. Examples of unpleasant pictures include bloody scenes, violent scenes, tragic scenes and harmful animals, such as cockroaches, snakes and spiders. Each picture was shown for 3 s, followed by an inter picture interval of 0.5 ± 0.2 s. An entire series of 100 pictures lasted 320–350 s.

#### 1.2.2. Short Standardized Film Fragments (SSFF)

A short film fragment was selected from the database of Gross and Levenson [36]. To induce state anxiety, we selected the basement chase scene (5′) from the movie *The Silence of the Lambs* [37], which is widely and successfully used to induce related anxiety mood states [38,39,40].

#### 1.2.3. Music Selection

Neutral music and happy music pieces were randomly selected from the Chinese Affective Music Mood System (CAMS), which provides a standard set of music stimuli for emotional research with Chinese participants [41]. Neutral music can induce soothing, pleasurable and calming emotions, and happy music can induce exciting and happy emotions. The music in the CAMS was selected by professional musicians and had a fixed loudness that was comfortable for participants. Six pieces of music were included for each musical emotional type (Neutral music: N06, N07, N09, N27, N38, N49; Happy music: H01, H02, H12, H14, H15, H46). Each piece of music was divided into 15 s, 30 s and 45 s in length, which has been shown to better activate emotion [42]. We excluded sad music because it may worsen subjects’ mood [43,44,45]. In addition, the blank stimulus (no sound) was used as a control condition.

#### 1.2.4. Questionnaire

The State-Trait Anxiety Inventory–State Anxiety scale (STAI-S) was administered to determine differences in subjects’ state anxiety levels before anxiety induction, after anxiety induction (before the anxiety intervention) and after the anxiety intervention. The STAI-S evaluated the subjects’ current state of anxiety by asking how they felt ‘at this moment’ using items that measure subjective feelings of tension, nervousness, worry and arousal. Internal consistency was high, with Cronbach’s α = 0.95. The State-Trait Anxiety Inventory-Trait Anxiety scale (STAI-T), the BFI and the TCSQ were completed before the experiment. The STAI-T assessed how subjects ‘generally feel’ about anxiety. The 20-item STAI-T scores range from 1 (not at all) to 4 (very much so), resulting in total scores ranging from 20 to 80, where higher scores indicate greater anxiety [46]. The BFI is a self-reported measurement tool designed to assess the high-order personality traits of extraversion, agreeableness, conscientiousness, neuroticism and openness [47]. The TCSQ assessed individual strategies against stress incidents [48].

### 1.3. Experimental Procedures and Tasks

**Figure 1:**
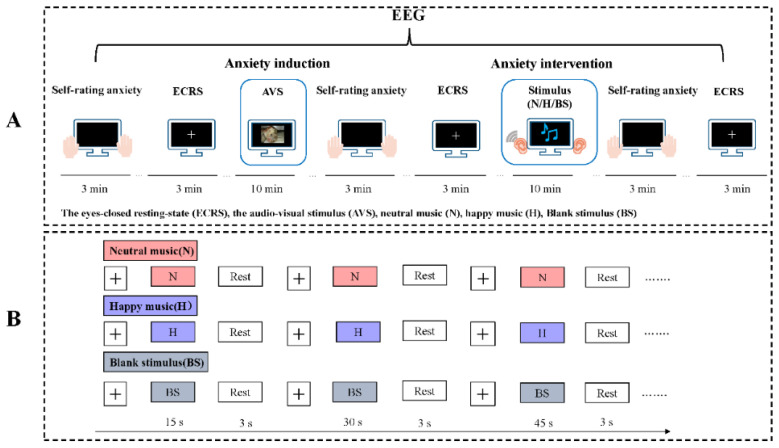
(**A**) Experimental paradigm. The experimental session had two phases: anxiety induction and anxiety intervention. Self-rated anxiety scores by scales and eyes-closed resting-state (ECRS) EEG signals were acquired before and after the two phases. In the anxiety induction phase, we used 100 negative target pictures (IAPS) and short standardized film fragments as audio-visual stimuli (AVSs) to evoke anxiety. In the anxiety intervention phase, the participants in each group received three interventions (neutral music stimulus, happy music stimulus and blank stimulus) after anxiety induction. (**B**) The anxiety intervention phase included the neutral music group, happy music group and blank stimulus group. The durations of the three interventions were 15 s, 30 s and 45 s, respectively. We selected six pieces of the same type of music and repeated the procedure six times with an interval of 3 s. The intervention lasted for 10 min in total. We set the volume for all the subjects at 50 decibels, but the subjects were allowed to adjust the volume if they found it to be too low or too high.

The experiment was divided into the following two stages: the anxiety induction phase and the anxiety intervention phase (Figure 1A).

In the anxiety induction phase, we used the evoked anxiety paradigm by autonomous design, which had been shown to have effects on 60 subjects in another experiment (30 males, 30 females; M ± SD age, 23.38 ± 1.46 y, Appendix A). Here, 62 subjects viewed 100 negative target pictures, which were selected from the IAPS, and watched the SSFF during a 10-minute period. The participants were informed that they would be asked 3 questions about the pictures and the video that they observed during the task to maintain their attention during the passive observation.

In the anxiety intervention phase, 62 subjects were randomly divided into the following three groups: the neutral music group (*n* = 21), happy music group (*n* = 21) and blank stimulus group (*n* = 20). The durations of the three interventions were 15 s, 30 s and 45 s, respectively, which have been shown to better activate emotion (Figure 1B). Each participant was seated in a comfortable chair in a sounded-attenuated, brightly lit, temperature-controlled (25–27 °C) room. The subjects were asked to carefully read the instructions on the screen and given earphones connected to a computer. We set the volume for all the subjects at 50 decibels, but the subjects were allowed to adjust it if they found it too low or too high. All participants were instructed to avoid strong head movements during the experiment.

EEG signals were acquired during the experiment. All subjects were asked to complete the STAI, and their resting-state EEGs were recorded with their eyes closed for 3 min before anxiety induction, after anxiety induction and after the anxiety intervention. E-prime 3.0 (Psychology Software Tools, Pittsburgh, PA, USA) was used to program the experimental tasks.

### 1.4. EEG Data Acquisition and Analyses

EEG recording was performed with 64 Ag/AgCl electrodes (ANT Neuro, Berlin, Germany). The electrodes were positioned according to the extended 10–20 system, and the data were recorded at a sampling rate of 1000 Hz. The bandpass filter was set at 0.3–100 Hz, and CPz was used as a reference electrode. Electrooculograms (EOGs) to monitor eye movements were recorded from an additional channel located on the left eye. During the entire experimental task, the impedance of the electrodes was maintained below 10 kΩ.

Resting-related EEG data were preprocessed with the following procedures. We first chose 61-channel EEG datasets and used independent component analysis to remove eye movement interference from raw EEG data. Then, the data were transformed to zero reference [49,50] using the EEGLAB (https://sccn.ucsd.edu/eeglab/index.php, accessed on 18 April 2021) toolbox REST (http://www.neuro.uestc.edu.cn/content/96, accessed on 18 April 2021) and filtered. Next, to reduce low-frequency drift and high-frequency noise, the re-referenced data sets were bandpass-filtered in the range of 1–50 Hz. Finally, a threshold of ± 100 μV was used to exclude fragments contaminated with high-amplitude artifacts.

#### 1.4.1. Power Spectrum Analysis

We analyzed the power spectral density (PSD) of the preprocessed EEG signals according to Welch theory [51] by windowing processing on the data through the selected window and then using the fast Fourier transform (FFT) to calculate the power spectrum and averages to obtain the relative value of the power spectrum density. Considering that the gamma band may play an important role in anxiety relief, we focused on the gamma band for the power spectrum analysis. Power spectrum amplitudes were calculated and compared across six conditions (neutral music: before the anxiety intervention vs. after the anxiety intervention; happy music: before the anxiety intervention vs. the after the anxiety intervention; blank stimulus: before the anxiety intervention vs. after the anxiety intervention) in the gamma band (35–45 Hz). Then, we calculated 2 regional brain segments, including the temporal lobe (T7, T8) and occipital lobe (O1, Oz, O2).

#### 1.4.2. Brain Functional Connectivity Analysis

The phase-locking value (PLV) is an alternative class of measures for functional networks that maintain constant phase separation between two signals (i.e., synchrony). In this study, we employed the phase-locking value to measure the cooperative, synchrony-defined neuronal assemblies at each frequency bin. The phase-locking value between signals *s*_1_(*t*) and *s*_2_(*t*) was formulized as follows.

First, we use the following Hilbert transform:

(1) $$z_{i}\left(t\right)=s_{i}\left(t\right)+jHT\left(s_{i}\left(t\right)\right)$$

Then, the constant phase between *z*_1_(*t*) and *z*_2_(*t*) by using analytical signals can be computed as,

(2) $$∆\emptyset \left(t\right)=arg\left(\frac{z_{1}\left(t\right)Z_{2}^{*}\left(t\right)}{\left|z_{1}\left(t\right)\right|\left|z_{2}\left(t\right)\right|}\right)$$

Finally, the instantaneous *PLV* is,

(3) $$PLV\left(t\right)=\left|E\left[e^{j∆\emptyset \left(t\right)}\right]\right|$$

Then, based on the phase-locking value constructed in the gamma band (35–45 Hz), we calculated the difference map of the phase-locking value in the conditions after anxiety induction and after the anxiety intervention.

### 1.5. Statistical Tests

ANOVA tests were used for comparison among before anxiety induction, after anxiety induction (before anxiety intervention) and after anxiety intervention in the condition of listening to neutral music and happy music. In the case of all analysis of variances, multiple comparisons were calculated using Bonferroni’s posttest. Wilcoxon matched-pairs signed-rank test was used when the assumptions of data normal distribution were not met in the condition of blank stimulus. ANOVA was used to compare state anxiety levels after anxiety induction among the neutral music group, happy music group and blank stimulus group. In the case of a normal distribution, we used a paired *t*-test to analyze changes in the power spectrum and phase-locking value before and after the anxiety intervention; otherwise, we used the Wilcoxon matched-pairs signed-rank test. Pearson’s correlations were calculated between the power spectrum and state anxiety. The Kruskal–Wallis test was used in cases of a nonnormal distribution for emotional intensity, arousal, pleasantness and dominance. One-sample *t*-tests were used in cases of a normal distribution for comparisons among neutral music, happy music in familiarity and trend degree. For correlations between personality traits, trait coping style and state anxiety after the anxiety intervention, Spearman’s correlations were used when data were not normally distributed, and Pearson’s correlations were used when data were normally distributed.

## 2. Results

### 2.1. Behavioral Alterations in Subjects with State Anxiety in Different Conditions

**Figure 2:**
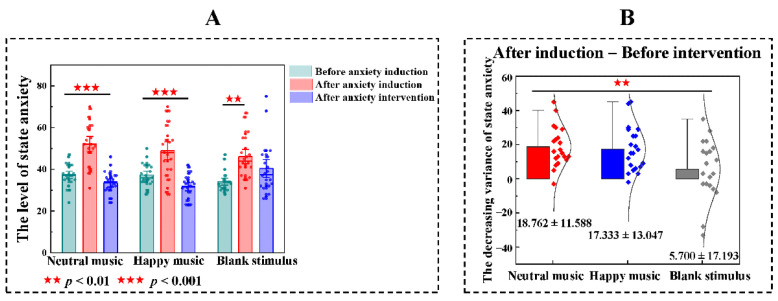
The effects of anxiety induction and the anxiety intervention. (**A**) Analysis of variance among before anxiety induction, after anxiety induction and after anxiety intervention in state anxiety scores. The level of state anxiety of three groups in after anxiety intervention are significantly higher than before anxiety induction. After anxiety intervention, neutral music group and happy music group were significantly lower than after anxiety induction. Notably, no significant difference between after anxiety induction and after anxiety intervention under blank stimulus. (**B**) The decreasing variance of state anxiety. After the anxiety intervention, compared with the decreasing variance of state anxiety in the blank stimulus group, the neutral music and happy music both showed the greater effect, but there was no significant difference between neutral music group and happy music group (after anxiety induction—after anxiety intervention).

ANOVA tests were used to make comparisons among the neutral music group (F = 35.224, *p* < 0.001) and the happy music group (F = 21.226, *p* < 0.001) before anxiety induction, after anxiety induction and after anxiety intervention. There were significant differences in two groups on the level of state anxiety (after anxiety induction > before anxiety induction >after anxiety intervention). The Wilcoxon matched-pairs signed-rank tests were used to compare before anxiety induction with after anxiety induction and compare after anxiety induction with after anxiety intervention in the blank stimulus. State anxiety scores of before anxiety induction were significantly lower than after anxiety induction (z = −3.462, *p* = 0.001), whereas state anxiety scores after anxiety induction and after the anxiety intervention were not significantly different in the blank stimulus group. ANOVA was also used for comparisons among the neutral music group, happy music group and blank stimulus group before and after anxiety induction. No significant differences in anxiety levels were observed among the three groups before or after induction (Figure 2A).

The ANOVA test also was used to make comparisons to the decreasing variance of state anxiety among the neutral music group, happy music group and blank stimulus group (F = 5.259, *p* = 0.008) and the Bonferroni’s posttest was used to make multiple comparisons among three groups. After the anxiety intervention, compared with the decreasing variance of state anxiety in the blank stimulus group (M ± SD = 5.700 ± 17.193), the neutral music (M ± SD = 18.762 ± 11.588, *p* = 0.013) and happy music (M ± SD = 17.333 ± 13.047, *p* = 0.031) both showed the greater effect, but there was no significant difference between neutral music group and happy music group (after anxiety induction—after anxiety intervention, Figure 2B).

### 2.2. Power Spectrum in Subjects with State Anxiety in Different Conditions

**Figure 3:**
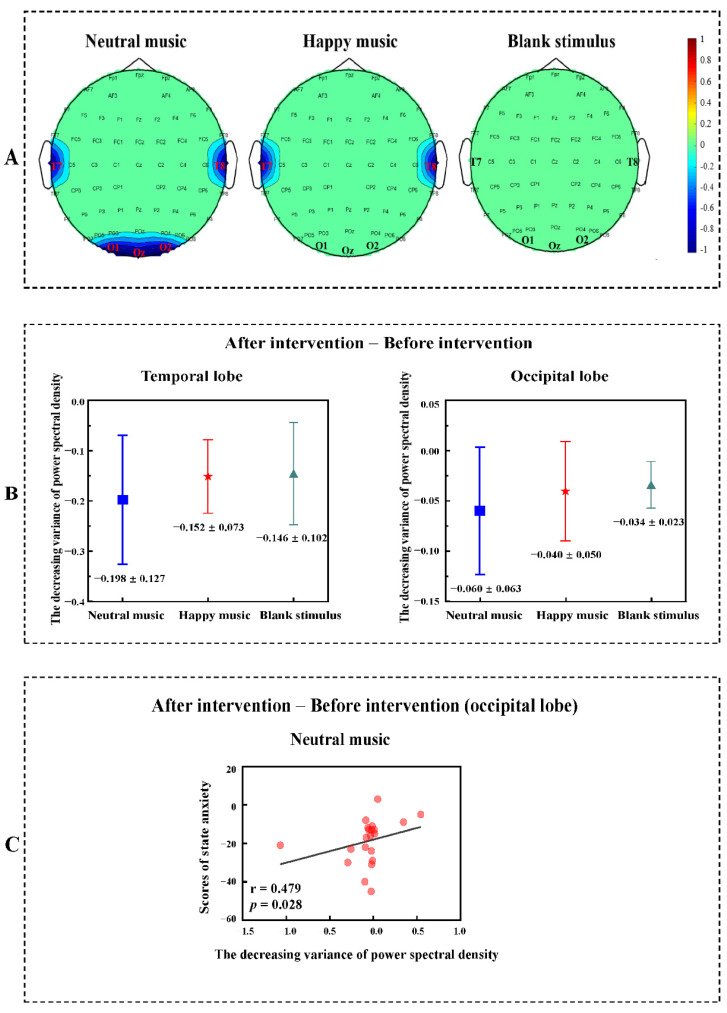
(**A**) Power spectrum differences before the anxiety intervention and after the anxiety intervention in the temporal lobe and occipital lobe in the gamma band (after anxiety intervention—before anxiety intervention). Blue represents reduced spectral power density. (**B**) The decreasing variance of power spectral density. There were no significant differences in the variance of power spectral density among the neutral music group, happy music group and blank stimulus group (after anxiety intervention—before anxiety intervention). (**C**) Correlation analyses between state anxiety and the power spectrum. Positive correlations were identified between state anxiety scores and power spectral density in the occipital lobe in the gamma band under the neutral music condition (after anxiety intervention—before anxiety intervention).

The power spectrum contrast (before anxiety intervention > after anxiety intervention) based on the Wilcoxon nonparametric *t*-test revealed decreased power spectral density after listening to neutral music in the temporal lobe (z = −2.172, *p* = 0.030) and occipital lobe (z = −2.103, *p* = 0.035) in the gamma band. The power spectrum contrast (before anxiety intervention > after anxiety intervention) based on the Wilcoxon nonparametric *t*-test revealed decreased power spectrum density in the happy music group (z = −2.277, *p* = 0.023) in the temporal lobe in the gamma band, but no significant difference was found in the occipital lobe in the gamma band. No difference was identified between the two brain regions in the blank stimulus group (Figure 3A).

For the temporal lobe, no significant differences in the variance of power spectral density were found among the neutral music group (M ± SEM = −0.198 ± 0.127), happy music group (M ± SEM = −0.152 ± 0.073), and blank stimulus group (M ± SEM = −0.146 ± 0.102). For the occipital lobe, there were also no significant differences in the variance of power spectral density among the neutral music group (M ± SEM = −0.060 ± 0.063), happy music group (M ± SEM = −0.040 ± 0.050), and blank stimulus group (M ± SEM = −0.034 ± 0.023, Figure 3B).

The state anxiety scores correlated positively with power spectral density in the occipital lobe when listening to neutral music (r = 0.479, *p* = 0.028, Figure 3C).

### 2.3. Brain Functional Connectivity between the Occipital Lobe and Other Lobes

**Figure 4:**
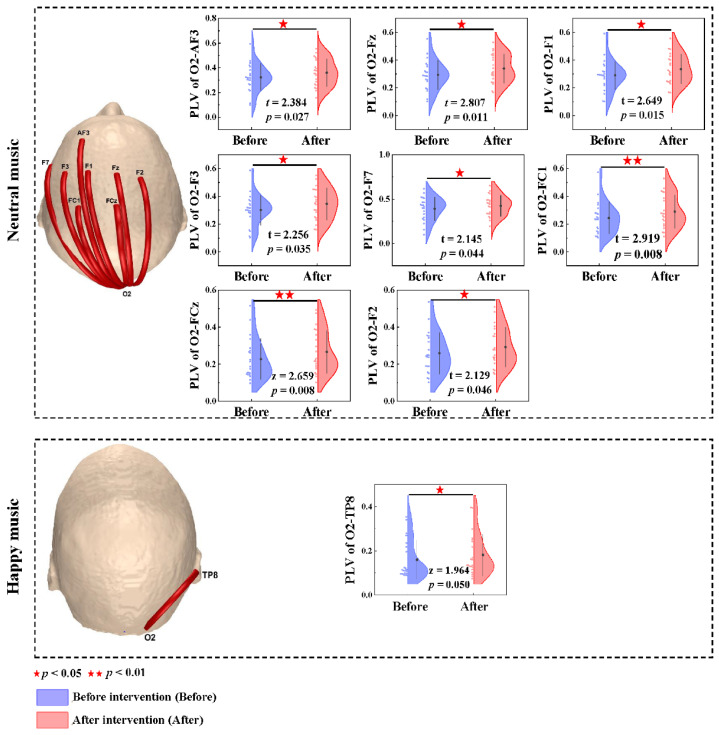
Brain functional connectivity in the whole brain and the phase-locking value differences before and after the anxiety intervention at the O_2_ electrode in the gamma band. Red lines denote enhanced functional connectivity. Blue and red half violins denote anxiety induction and anxiety intervention, respectively.

The occipital lobe was demonstrated to be a significant predictor of the effect of the anxiety music intervention based on the results presented in the previous section. Hence, the occipital cortex emerged as the preferred region, and phase-locking values between the occipital lobe and the remaining brain regions were computed. The phase-locking value contrast (before anxiety intervention < after anxiety intervention) based on the paired *t*-test and Wilcoxon nonparametric *t*-test revealed increased brain functional connectivity after listening to neutral music at the O2-AF3 (t = 2.384, *p* = 0.027), O2-Fz (t = 2.807, *p* = 0.011), O2-F1 (t = 2.649, *p* = 0.015), O2-F3 (t = 2.256, *p* = 0.035), O2-F7 (t = 2.145, *p* = 0.044), O2-FC1 (t = 2.919, *p* = 0.008), O2-FCz (z = 2.659 *p* = 0.008) and O2-F2 (t = 2.129, *p* = 0.046) channels in the gamma band. The phase-locking value in the happy music group based on the Wilcoxon nonparametric *t*-test revealed increased brain functional connectivity at the O2-TP8 (z = 1.964, *p* = 0.050) channel in the gamma band (Figure 4).

### 2.4. Analysis of Musical Elements, Personality and Coping Style

**Figure 5:**
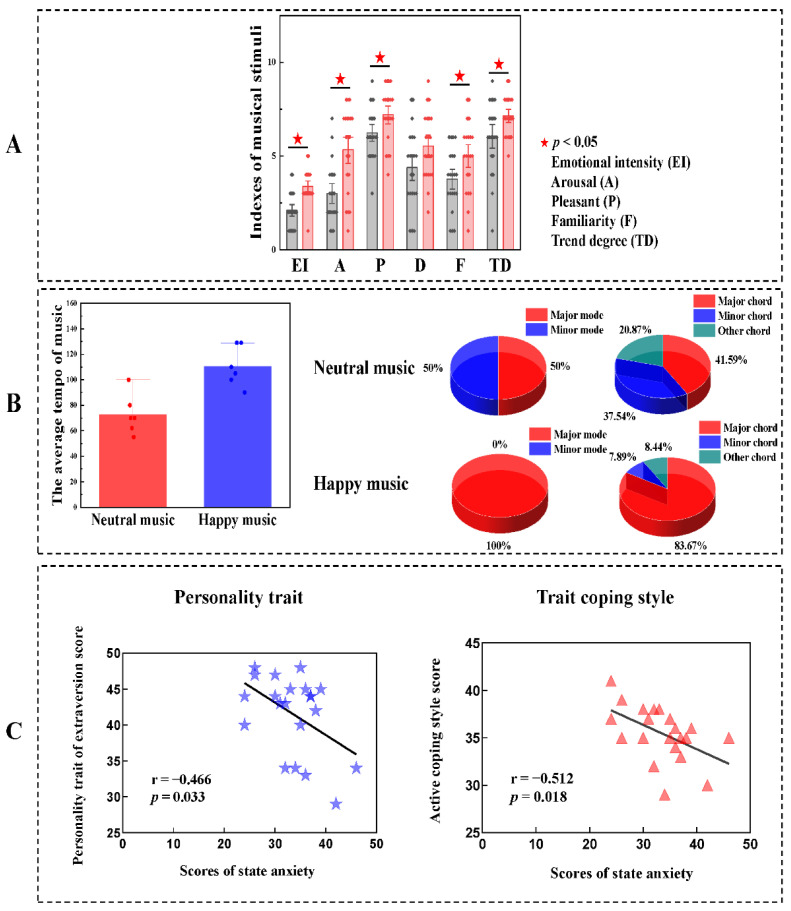
Analysis of musical elements, personality and coping style. (**A**) Analysis of variance of the scores for affective valences between the neutral music group and the happy music group. (**B**) Differences in characteristics between neutral music and happy music. The average tempo of neutral music was slower than that of happy music. For neutral music, major mode accounted for 50% of the musical sequence, minor mode accounted for 50% of the musical sequence, major chord accounted for 41.59% of the musical sequence, minor chord accounted for 37.54% of the musical sequence and other chord accounted for 20.87% of the musical sequence. For happy music, major mode accounted for 100% of the musical sequence, major chord accounted for 83.67% of the musical sequence, minor chord accounted for 7.89% of the musical sequence and other chord accounted for 8.44% of the musical sequence. (**C**) Correlation analyses between personality traits, trait coping style and state anxiety after the neutral music intervention. Negative correlations were found between state anxiety scores and personality trait extraversion and active coping style when listening to neutral music.

The scores of the indexes of emotional intensity (EI, z = −3.887, *p* < 0.001), arousal (A, z = −3.209, *p* = 0.001), pleasantness (P, z = −2.142, *p* = 0.032), familiarity (F, t = −2.280, *p* = 0.028) and trend degree (TD, t = −2.297, *p* = 0.028) in the neutral music group were significantly lower than those in the happy music group. No significant difference in dominance was observed between the two groups (Figure 5A). The average tempo of neutral music ranged from 55 to 100 bpm (M ± SD = 72.833 ± 15.753), which was lower than that of happy music, which ranged from 90 to 129 bpm (M ± SD = 11.500 ± 15.783). For neutral music, major mode accounted for 50% of the musical sequence, minor mode accounted for 50% of the musical sequence, major chord accounted for 41.59% of the musical sequence, minor chord accounted for 37.54% of the musical sequence and other chord accounted for 20.87% of the musical sequence. For happy music, major mode accounted for 100% of the musical sequence, major chord accounted for 83.67% of the musical sequence, minor chord accounted for 7.89% of the musical sequence and other chord accounted for 8.44% of the musical sequence (Figure 5B). Considering individual differences, we also correlated trait personality and trait coping style with state anxiety after listening to neutral music. The state anxiety scores were significantly negatively correlated with the personality trait extraversion (r = −0.466, *p* = 0.033) and an active coping style (r = −0.512, *p* = 0.018) in the neutral music group (Figure 5C).

## 3. Discussion

Our results indicated that neutral music and happy music both had the effect of state anxiety intervention. Moreover, neutral music ameliorating state anxiety was associated with a decreased power spectral density of the occipital lobe and increased brain functional connectivity between the occipital lobe and frontal lobe. Happy music ameliorating state anxiety was associated with the increased brain functional connectivity between the occipital lobe and right temporal lobe. These findings may extend the current understanding of state anxiety.

### 3.1. Music Intervention Has the Effect When Coupled with an Anxiety Intervention: A Perspective from the Musical Elements, Personality and Coping Style

Prior studies on anxiety have identified that listening to prerecorded music may have a beneficial effect on preoperative anxiety [12]. Listening to both neutral and happy music reduced subjects’ anxiety [13,14]. Here, by setting three groups (neutral music, happy music and blank stimulus) together, we found that the neutral music group and happy music group both showed better effects than blank stimulus group after the anxiety intervention.

Even though the anxiety reductions were similar for the two types of music, there were fundamental differences between these types of music. Compared with happy music, neutral music has a lower average tempo (Appendix B). A study showed that slow music had a better effect on anxiety than fast music, which is consistent with our results [52]. Moreover, we found that the proportions of both mode and chord in neutral music were more balanced than happy music. Happy music has more popular chords than neutral music, which are always used in many popular songs. The chords and pitches in neutral music often deviate from modes, which is a common technique to add more emotion and tension to songs. Musical modes and chords have well-established emotional resonance [53].Multiple modes or chords may alleviate anxiety more than one mode or chord alone. Music may have played a specific biological role in the existence of a distinctive brain knowledge system, and some elements of music knowledge (such as melodies) may be meaningful to the study of science [54]. The anterior portion of the bilateral temporal lobes is vital in the discrimination of melodies and chords [55]. When hearing musical sounds, such as chords, subjects might show unique patterns of connectivity between temporal and occipital lobe regions [56]. The results of our study are in accordance with these results. In addition, emotion from music is another important factor in anxiety interventions. Emotional intensity, arousal, pleasantness, familiarity and trend degrees associated with neutral music were significantly lower than those associated with happy music. These elements of neutral music illustrate that subjects listening to both relaxing and calm music may be satisfied and happy during anxiety interventions.

The morphology and function of the medial prefrontal cortex (mPFC) and the dorsal–lateral prefrontal cortex (dlPFC) in particular were associated with individual differences in trait personalities with anxiety [57,58,59]. Personality plays an important role in the modulation of state anxiety. Inferior frontal areas may also be implicated in apathy, compulsive behaviors and changes in personality and social deficits [60]. Higher scores on the extraversion personality trait were accompanied by less anxiety induced by neutral stimuli with neutral music. The role of personality is a mediator between depressive mood and mortality [61]. Personality acted as a mediator between music and anxiety. The individuals who were skilled at interpersonal interactions in the majority groups lessened anxiety-like behaviors. Subjects with extraversion personality have a relatively larger capacity of emotion than other trait personalities. In addition, coping style, which is known to impact psychological well-being, may serve as a control for variables in future studies [62]. The individuals with positive coping styles under the condition of neutral music reduced the risk of state anxiety. Such positive reinterpretation is not only a means to reduce emotional distress but also a form of active, problem-focused coping [63].

### 3.2. Music Interventions in Anxiety Correlate with Decreased Power Spectral Density of the Occipital Lobe

An fMRI study used visual-to-music sensory substitution to study the processing of numerical symbols and letters in congenitally blind individuals [64]. Auditory stimuli can activate the visual cortex [54]. These studies indicate that auditory stimuli are associated with the visual cortex. In our study, the power spectrum could reflect decreased power spectral density at the occipital lobe in the gamma band under the condition of neutral music. A study provided further support for the involvement of anterior temporal and occipital cortical regions during anticipatory anxiety and suggested that both excitatory and inhibitory processes were associated with anticipatory anxiety alterations [65]. Our results were consistent with the study mentioned above, which demonstrated that low state anxiety was associated with a decrease in power spectral density in the temporal lobe and occipital lobe after neutral music intervention. Further detailed investigations of the interplay between music and anxiety interventions confirmed the important role of the occipital lobe. Previous studies have suggested that the patterns of occipital lobe activity were sufficient for decoding emotion categories evoked by images and emotional states elicited by cinematic movies [66]. The process of musical emotion may be associated with the occipital lobe. The acoustic signals might be enhanced in the occipital cortex at the gamma band to evoke neutral and relaxing emotions. The neutral and relaxing emotions evoked by neutral music prevented increasing state anxiety. According to the musical affective valence and musical characteristics, neutral music exhibited a lower affective valence, a slower rhythm and smoother tempo. These contact characteristics may have been important factors in anxiety intervention by music. One recent study revealed that natural sounds improve health, increase positive affect and lower stress and annoyance [67], which may be consistent with the emotional tone of neutral music in our study.

In addition, both the neutral music group and the happy music group maintained decreased the power spectral density of the temporal lobe. Temporal lobe structures include the amygdala and the hippocampus, the right amygdala is robustly associated with negative emotion, particularly in fear [68], and positive stimuli activate the left amygdala through the brain’s reward system [69]. Neutral and happy music intervention improved state anxiety to different extents by the interactions of positive and negative emotion. The interactions of emotion may exist inside and not be associated with acoustic signals.

### 3.3. State Anxiety Intervention by Different Emotional Music Has Different Brain Functional Connectivity

Another finding further showed that the brain functional connectivity between the occipital lobe and frontal lobe would increase after neutral music intervention. Connectivity of the PFC is a locus that is also associated with mood regulation. Anxious participants showed a larger asymmetry in favor of the hemisphere than that of the control group [69]. Negative emotion has been linked to the medial frontal cortex, including the anterior midcingulate cortex [70]. Individuals with high anxiety or an anxiety disorder had decreased functional connectivity changes associated with the network of the frontal lobe in tasks [71]. The control group showed significantly more prefrontal activation and less activation in the occipital lobes than the autism spectrum disorder group during emotional face matching [72]. Our results were consistent with this finding. Through power spectra analysis, the occipital cortex emerged as our preferred region, and we found that the brain functional connectivity between the occipital lobe and frontal lobe increased after neutral music intervention. Furthermore, the medial prefrontal cortex interacts with both the ventral hippocampus and the amygdala and may modulate the anxiety state based on previous experiences or internal needs of the animal [73]. However, which region in the PFC has a direct link with anxiety under stressed conditions is often unclear. An electroencephalography study explored how the push–pull interaction between top-down and bottom-up attention manifests itself in dynamic auditory scenes, and a controlled rhythmic sound sequence significantly suppressed phase-locking and gamma responses to the attended sequence, countering enhancement effects observed for attended targets [74]. Anxiety is related to enhancing activation of the ventral attention networks [72]. Attention can change the brain when music acts as an acoustic stimulus in the process of anxiety intervention. Our results indicate a possible brain mechanical pathway between neutral music and anxiety. When we listened to neutral music, our attentional resources were occupied and developed bottom-up attention. In addition, brain functional connectivity increased between the occipital lobe and right parietal-temporal lobe after happy music intervention. High anxiety is also associated with increased connectivity between the amygdala and distributed brain systems, which are involved in attention, emotion perception and regulation, and these effects are most prominent in the basolateral amygdala [75]. The right hemisphere has a special role in emotion in the intact brain [76], and happy intonations lead to right-lateralized activation in the temporal lobe [77], which illustrates that the right temporal lobe may specialize in happy emotional processing after listening to happy music. Under the condition of shifting attention, empathy may automatically participate [78] and further deepen the intervention of neutral emotion on anxiety. For happy music intervention, the change in brain function mainly originated from the happiness emotion and anxiety trading off with each other. For neutral music intervention, the change in brain function might mainly originate from emotion, attention and empathy working together. This strongly indicates the interaction with attention. This finding suggests that brain functional connectivity between the occipital lobe and frontal lobe might be a unique therapeutic target for using neutral music to treat state anxiety.

This study has some limitations. First, patients with anxiety disorders should be recruited to determine the therapeutic effect of neutral music on anxiety in the future. Second, only two emotional types of music were selected in this experiment. Whether other types of music can ameliorate anxiety and the associated neural mechanisms remain to be further explored. In addition, correction for multiple comparisons was not performed with respect to the testing of multiple channels and groups of EEG channels, so our findings should be considered exploratory rather than confirmatory. Finally, further studies should also consider attentional and emotional effects on state anxiety modulation by music. 

## 4. Conclusions

In conclusion, neutral music and happy music had better effects on alleviating state anxiety than blank stimulus. The brain mechanisms supported that neutral music ameliorating state anxiety was associated with a decreased gamma power spectral density of the occipital lobe. Neutral music ameliorating state anxiety was associated with an increased brain functional connectivity between the occipital lobe and frontal lobe, which might have been caused by bottom-up auditory attention and enhanced emotional blunting in the relatively narrow rhythm. For happy music, the brain mechanism was associated with enhanced brain functional connectivity between the occipital lobe and right temporal lobe, which might simply depend on a trade-off between happy emotions and anxious emotions. Our findings also suggested that musical elements, trait personality and trait coping style played important roles in the anxiety intervention. This study may be important for deeply understanding the mechanisms evoked by state anxiety music interventions and may contribute to clinical treatment with nonpharmaceutical interventions in the future.

## Figures and Tables

**Figure A1:**
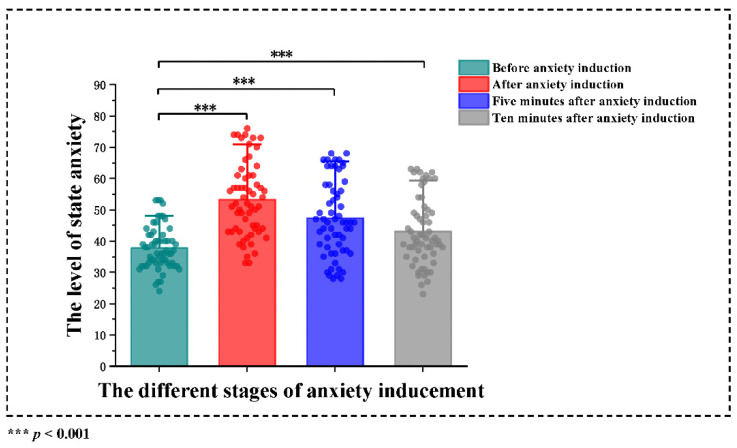
The effect of the anxiety-induced paradigm.

## Data Availability

The datasets generated for this study are available on request to the corresponding author.

## Appendix A

To test the efficacy of anxiety induction, the STAI was used to evaluate state anxiety before anxiety induction, after anxiety induction, 5 min after anxiety induction and 10 min after anxiety induction. Wilcoxon matched-pairs signed-rank tests among before anxiety induction, after anxiety induction, five minutes after anxiety induction and 10 minutes after anxiety induction in state anxiety. The level of state anxiety before induction was significantly lower than those after anxiety induction (z = −6.167, *p* < 0.001), five minutes after anxiety induction (z = −4.625, *p* < 0.001) and 10 minutes after anxiety induction (z = −2.790, *p* = 0.005).

## Appendix B

**Table A1:** Analysis of musical elements.

Music	Tempo	Mode	Chord
N06	55	D	Major chord: 50% Minor chord: 50% Other chord: 0%
N07	70	Am	Major chord: 25%; Minor chord: 75% Other chord: 0%
N09	80	E	Major chord: 16.67% Minor chord: 0% Other chord: 83.3%
N27	70	D	Major chord: 66.67% Minor chord: 20.83% Other chord: 12.5%
N38	100	#Fm	Major chord: 50% Minor chord: 50% Other chord: 0%
N49	62	#Cm	Major chord: 41.17% Minor chord: 29.41% Other chord: 29.41%
Average of all neutral music	72.83	Major mode: 50% Minor mode: 50%	Major chord: 41.59% Minor chord: 37.54% Other chord: 20.87%
H01	100	G	Major chord: 77.08% Minor chord: 22.92% Other chord: 0%
H02	105	E	Major chord: 92.31% Minor chord: 0% Other chord: 7.69%
H12	110	C	Major chord: 100% Minor chord: 0% Other chord: 0%
H14	90	D	Major chord: 68.89% Minor chord: 24.44% Other chord: 6.67%
H15	129	G	Major chord: 66.67% Minor chord: 0% Other chord: 33.33%
H46	129	#F	Major chord: 97.06% Minor chord: 0% Other chord: 2.94%
Average of all happy music	110.5	Major mode: 100% Minor mode: 0%	Major chord: 83.67% Minor chord: 7.89% Other chord: 8.44%

We analyzed each piece of music through music theory and deduced each bar (half bar or quarter bar if necessary). Then, they were tested by at least three musicians or composers to ensure the accuracy of the analysis. The letters in mode represent the mode (major or minor) of the piece of music, where the corner mark m represents minor, and vice versa. For example, the mode of N06 is D, which means that the mode of N06 is D major mode. The mode of N07 is Am, which means that the mode of N07 is A minor mode. The proportions of major and minor chords are calculated in terms of how long they are in the audio.

## References

[1] Gross C., Hen R. (2004). The developmental origins of anxiety. Nat. Rev. Neurosci..

[2] De Gregorio D., McLaughlin R.J., Posa L., Ochoa-Sanchez R., Enns J., Lopez-Canul M., Aboud M., Maione S., Comai S., Gobbi G. (2019). Cannabidiol modulates serotonergic transmission and reverses both allodynia and anxiety-like behavior in a model of neuropathic pain. Pain.

[3] Makovac E., Smallwood J., Watson D.R., Meeten F., Critchley H.D., Ottaviani C. (2018). The verbal nature of worry in generalized anxiety: Insights from the brain. NeuroImage Clin..

[4] Alvaro P.K., Roberts R.M., Harris J.K. (2013). A Systematic Review Assessing Bidirectionality between Sleep Disturbances, Anxiety, and Depression. Sleep.

[5] Jacobson N.C., Newman M.G. (2017). Anxiety and depression as bidirectional risk factors for one another: A meta-analysis of longitudinal studies. Psychol. Bull..

[6] Taylor P.J., Gooding P., Wood A.M., Tarrier N. (2011). The Role of Defeat and Entrapment in Depression, Anxiety, and Suicide. Psychol. Bull..

[7] Stuber G.D., Mason A.O. (2013). Integrating optogenetic and pharmacological approaches to study neural circuit function: Current applications and future directions. Pharmacol. Rev..

[8] Bahrke M.S., Morgan W.P. (1978). Anxiety reduction following exercise and meditation. Cognit. Ther. Res..

[9] Compton S.N., March J.S., Brent D., Albano A.M., Weersing V.R., Curry J. (2004). Cognitive-behavioral psychotherapy for anxiety and depressive disorders in children and adolescents: An evidence-based medicine review. J. Am. Acad. Child Adolesc. Psychiatry.

[10] Lin C.-J., Chang Y.-C., Chang Y.-H., Hsiao Y.-H., Lin H.-H., Liu S.-J., Chao C.-A., Wang H., Yeh T.-L. (2019). Music Interventions for Anxiety in Pregnant Women: A Systematic Review and Meta-Analysis of Randomized Controlled Trials. J. Clin. Med..

[11] Chlan L.L., Weinert C.R., Heiderscheit A., Tracy M.F., Skaar D.J., Guttormson J.L., Savik K. (2013). Effects of Patient-Directed Music Intervention on Anxiety and Sedative Exposure in Critically Ill Patients Receiving Mechanical Ventilatory Support: A Randomized Clinical Trial. JAMA.

[12] Bradt J., Dileo C., Shim M. (2013). Music interventions for preoperative anxiety. Cochrane Database Syst. Rev..

[13] Riegler S.J., Miller R. (1980). Degrees of familiar and affective music and their effects on state anxiety. J. Music Ther..

[14] Hodges D.A. (2008). Bodily responses to music. Oxford Handbook of Music Psychology.

[15] Felix-Ortiz A.C., Beyeler A., Seo C., Leppla C.A., Wildes C.P., Tye K.M. (2013). BLA to vHPC inputs modulate anxiety-related behaviors. Neuron.

[16] Stujenske J.M., Likhtik E., Topiwala M.A., Gordon J.A. (2014). Fear and Safety Engage Competing Patterns of Theta-Gamma Coupling in the Basolateral Amygdala. Neuron.

[17] Bi K., Chattun M.R., Liu X., Wang Q., Tian S., Zhang S., Lu Q., Yao Z. (2018). Abnormal early dynamic individual patterns of functional networks in low gamma band for depression recognition. J. Affect. Disord..

[18] Apps R., Strata P. (2015). Neuronal circuits for fear and anxiety-the missing link. Nat. Rev. Neurosci..

[19] Reiman E.M., Fusselman M.J., Fox P.T., Raichle M.E. (1989). Neuroanatomical correlates of anticipatory anxiety. Science.

[20] Hasler G., Fromm S., Alvarez R.P., Luckenbaugh D.A., Drevets W.C., Grillon C. (2007). Cerebral blood flow in immediate and sustained anxiety. J. Neurosci..

[21] Schienle A., Schäfer A., Stark R., Walter B., Vaitl D. (2005). Relationship between disgust sensitivity, trait anxiety and brain activity during disgust induction. Neuropsychobiology.

[22] Seo D., Ahluwalia A., Potenza M.N., Sinha R. (2017). Gender differences in neural correlates of stress-induced anxiety. J. Neurosci. Res..

[23] Menon V., Levitin D.J. (2005). The rewards of music listening: Response and physiological connectivity of the mesolimbic system. Neuroimage.

[24] Koelsch S., Fritz T., Cramon D.Y.V., Müller K., Friederici A.D. (2006). Investigating emotion with music: An fMRI study. Hum. Brain Mapp..

[25] Limb C.J. (2006). Structural and functional neural correlates of music perception. Anat. Rec. Part A Discov. Mol. Cell. Evol. Biol..

[26] Blenkmann A.O., Collavini S., Lubell J., Llorens A., Funderud I., Ivanovic J., Larsson P.G., Meling T.R., Bekinschtein T., Kochen S. (2019). Auditory deviance detection in the human insula: An intracranial EEG study. Cortex.

[27] Seymour R.A., Rippon G., Gooding-Williams G., Sowman P.F., Kessler K. (2020). Reduced auditory steady state responses in autism spectrum disorder. Mol. Autism.

[28] Sivarao D.V., Chen P., Senapati A., Yang Y., Fernandes A., Benitex Y., Whiterock V., Li Y.-W., Ahlijanian M.K. (2016). 40 Hz auditory steady-state response is a pharmacodynamic biomarker for cortical NMDA receptors. Neuropsychopharmacology.

[29] Schmitgen A., Saal J., Sankaran N., Desai M., Joseph I., Starr P., Chang E.F., Shirvalkar P. (2021). Musical Hallucinations in Chronic Pain: The Anterior Cingulate Cortex Regulates Internally Generated Percepts. Front. Neurol..

[30] Clare E.L., Holderied M.W. (2015). Acoustic shadows help gleaning bats find prey, but may be defeated by prey acoustic camouflage on rough surfaces. Elife.

[31] Bastos A.M., Usrey W.M., Adams R.A., Mangun G.R., Fries P., Friston K.J. (2012). Canonical Microcircuits for Predictive Coding. Neuron.

[32] Tanaka S., Kirino E. (2017). Dynamic reconfiguration of the supplementary motor area network during imagined music performance. Front. Hum. Neurosci..

[33] Orndorff-Plunkett F., Singh F., Aragón O.R., Pineda J.A. (2017). Assessing the effectiveness of neurofeedback training in the context of clinical and social neuroscience. Brain Sci..

[34] Bradley M., Lang P. (2007). The International Affective Picture System (IAPS). Handbook of Emotion Elicitation and Assessment.

[35] Lang P., Bradley M., Cuthbert B. (2005). International Affective Picture System (IAPS): Digitized Photographs, Instruction Manual and Affective Ratings. Tech. Rep..

[36] Gross J.J., Levenson R.W. (1995). Emotion Elicitation using Films. Cogn. Emot..

[37] Jungkunz V.G. (2016). The Silence of the Lambs.

[38] Marzillier S.L., Davey G.C.L. (2005). Anxiety and disgust: Evidence for a unidirectional relationship. Cogn. Emot..

[39] Macht M., Mueller J. (2007). Immediate effects of chocolate on experimentally induced mood states. Appetite.

[40] Converse B.A., Lin S., Keysar B., Epley N. (2008). In the Mood to Get Over Yourself: Mood Affects Theory-of-Mind Use. Emotion.

[41] Cheng J., Jiao C., Luo Y., Cui F. (2017). Music induced happy mood suppresses the neural responses to other’s pain: Evidences from an ERP study. Sci. Rep..

[42] Janata P., Grafton S.T. (2003). Swinging in the brain: Shared neural substrates for behaviors related to sequencing and music. Nat. Neurosci..

[43] ter Bogt T., Canale N., Lenzi M., Vieno A., van den Eijnden R. (2021). Sad music depresses sad adolescents: A listener’s profile. Psychol. Music.

[44] Wagener G.L., Berning M., Costa A.P., Steffgen G., Melzer A. (2021). Effects of Emotional Music on Facial Emotion Recognition in Children with Autism Spectrum Disorder (ASD). J. Autism Dev. Disord..

[45] Wilhelm K., Gillis I., Schubert E., Whittle E.L. (2013). On a Blue Note: Depressed Peoples’ Reasons for Listening to Music. Music Med..

[46] Spielberger C.D. (2010). State-Trait Anxiety Inventory. Corsini Encycl. Psychol..

[47] Rammstedt B., John O.P. (2017). Big Five Inventory. Encycl. Personal. Individ. Differ..

[48] Folkman S., Lazarus R.S. (1988). Coping as a Mediator of Emotion. J. Pers. Soc. Psychol..

[49] Yao D. (2001). A method to standardize a reference of scalp EEG recordings to a point at infinity. Physiol. Meas..

[50] Dong L., Li F., Liu Q., Wen X., Lai Y., Xu P., Yao D. (2017). MATLAB toolboxes for reference electrode standardization technique (REST) of scalp EEG. Front. Neurosci..

[51] Welch P.D. (1967). The Use of Fast Fourier Transform for the Estimation of Power Spectra: A Method Based on Time Averaging Over Short, Modified Periodograms. IEEE Trans. Audio Electroacoust..

[52] Rane P.R., Gadkari J.V. (2017). The effect of slow and fast musical tempo on post-exercise recovery on recovery period in young adults. Natl. J. Physiol. Pharm. Pharmacol..

[53] Agustus J.L., Mahoney C.J., Downey L.E., Omar R., Cohen M., White M.J., Scott S.K., Mancini L., Warren J.D. (2015). Functional MRI of music emotion processing in frontotemporal dementia. Ann. N. Y. Acad. Sci..

[54] Omar R., Hailstone J.C., Warren J.E., Crutch S.J., Warren J.D. (2010). The cognitive organization of music knowledge: A clinical analysis. Brain.

[55] Satoh M., Takeda K., Nagata K., Hatazawa J., Kuzuhara S. (2003). The Anterior Portion of the Bilateral Temporal Lobes Participates in Music Perception: A Positron Emission Tomography Study. Am. J. Neuroradiol..

[56] Zamm A., Schlaug G., Eagleman D.M., Loui P. (2013). Pathways to seeing music: Enhanced structural connectivity in colored-music synesthesia. Neuroimage.

[57] Takizawa R., Nishimura Y., Yamasue H., Kasai K. (2014). Anxiety and performance: The disparate roles of prefrontal subregions under maintained psychological stress. Cereb. Cortex.

[58] Indovina I., Robbins T.W., Núñez-Elizalde A.O., Dunn B.D., Bishop S.J. (2011). Fear-Conditioning Mechanisms Associated with Trait Vulnerability to Anxiety in Humans. Neuron.

[59] Heinz A., Braus D.F., Smolka M.N., Wrase J., Puls I., Hermann D., Klein S., Grüsser S.M., Flor H., Schumann G. (2005). Amygdala-prefrontal coupling depends on a genetic variation of the serotonin transporter. Nat. Neurosci..

[60] Yau Y., Zeighami Y., Baker T.E., Larcher K., Vainik U., Dadar M., Fonov V.S., Hagmann P., Griffa A., Mišić B. (2018). Network connectivity determines cortical thinning in early Parkinson’s disease progression. Nat. Commun..

[61] Lemogne C., Nabi H., Zins M., Cordier S., Ducimetière P., Goldberg M., Consoli S.M. (2010). Hostility may explain the association between depressive mood and mortality: Evidence from the french gazel cohort study. Psychother. Psychosom..

[62] Sun C.L., Francisco L., Baker K.S., Weisdorf D.J., Forman S.J., Bhatia S. (2011). Adverse psychological outcomes in long-term survivors of hematopoietic cell transplantation: A report from the bone marrow transplant survivor study (BMTSS). Blood.

[63] Park S., Choi J., Lee S., Oh C., Kim C., La S., Lee J., Suh B. (2019). Designing a chatbot for a brief motivational interview on stress management: Qualitative case study. J. Med. Internet Res..

[64] Abboud S., Maidenbaum S., Dehaene S., Amedi A. (2015). A number-form area in the blind. Nat. Commun..

[65] Gray M., Kemp A.H., Silberstein R.B., Nathan P.J. (2003). Cortical neurophysiology of anticipatory anxiety: An investigation utilizing steady state probe topography (SSPT). Neuroimage.

[66] Kragel P.A., Reddan M.C., LaBar K.S., Wager T.D. (2019). Emotion schemas are embedded in the human visual system. Sci. Adv..

[67] Buxton R.T., Pearson A.L., Allou C., Fristrup K., Wittemyer G. (2021). A synthesis of health benefits of natural sounds and their distribution in national parks. Proc. Natl. Acad. Sci. USA.

[68] Lanteaume L., Khalfa S., Régis J., Marquis P., Chauvel P., Bartolomei F. (2007). Emotion induction after direct intracerebral stimulations of human amygdala. Cereb. Cortex.

[69] Murray E.A., Izquierdo A., Malkova L. (2009). Amygdala Function in Positive Reinforcement: Contributions from Studies of Nonhuman Primates.

[70] Heller W., Nitschke J.B., Etienne M.A., Miller G.A. (1997). Patterns of regional brain activity differentiate types of anxiety. J. Abnorm. Psychol..

[71] Kragel P.A., Kano M., Van Oudenhove L., Ly H.G., Dupont P., Rubio A., Delon-Martin C., Bonaz B.L., Manuck S.B., Gianaros P.J. (2018). Generalizable representations of pain, cognitive control, and negative emotion in medial frontal cortex. Nat. Neurosci..

[72] Sylvester C.M., Corbetta M., Raichle M.E., Rodebaugh T.L., Schlaggar B.L., Sheline Y.I., Zorumski C.F., Lenze E.J. (2012). Functional network dysfunction in anxiety and anxiety disorders. Trends Neurosci..

[73] Kleinhans N.M., Richards T., Weaver K., Johnson L.C., Greenson J., Dawson G., Aylward E. (2010). Association between amygdala response to emotional faces and social anxiety in autism spectrum disorders. Neuropsychologia.

[74] Huang N., Elhilali M. (2020). Push-pull competition between bottom-up and top-down auditory attention to natural soundscapes. Elife.

[75] Qin S., Young C.B., Duan X., Chen T., Supekar K., Menon V. (2014). Amygdala subregional structure and intrinsic functional connectivity predicts individual differences in anxiety during early childhood. Biol. Psychiatry.

[76] Schwartz G.E., Davidson R.J., Maer F. (1975). Right hemisphere lateralization for emotion in the human brain: Interactions with cognition. Science.

[77] Sander K., Roth P., Scheich H. (2003). Left-lateralized fMRI activation in the temporal lobe of high repressive women during the identification of sad prosodies. Cogn. Brain Res..

[78] Morelli S.A., Lieberman M.D. (2013). The role of automaticity and attention in neural processes underlying empathy for happiness, sadness, and anxiety. Front. Hum. Neurosci..

